# *Rubinisphaera italica* sp. nov. isolated from a hydrothermal area in the Tyrrhenian Sea close to the volcanic island Panarea

**DOI:** 10.1007/s10482-019-01329-w

**Published:** 2019-11-26

**Authors:** Nicolai Kallscheuer, Mareike Jogler, Sandra Wiegand, Stijn H. Peeters, Anja Heuer, Christian Boedeker, Mike S. M. Jetten, Manfred Rohde, Christian Jogler

**Affiliations:** 1 Department of Microbiology, Radboud Universiteit Nijmegen, Nijmegen, The Netherlands; 2 Leibniz Institute DSMZ, Braunschweig, Germany; 3 Central Facility for Microscopy, Helmholtz Centre for Infection Research, HZI, Braunschweig, Germany

**Keywords:** Marine bacteria, Planctomycetes, Algae, Hydrothermal area, Panarea

## Abstract

*Planctomycetes* is a fascinating phylum of mostly aquatic bacteria, not only due to the environmental importance in global carbon and nitrogen cycles, but also because of a unique cell biology. Their lifestyle and metabolic capabilities are not well explored, which motivated us to study the role of Planctomycetes in biofilms on marine biotic surfaces. Here, we describe the novel strain Pan54^T^ which was isolated from algae in a hydrothermal area close to the volcanic island Panarea in the Tyrrhenian Sea, north of Sicily in Italy. The strain grew best at pH 9.0 and 26 °C and showed typical characteristics of planctomycetal bacteria, e.g. division by polar budding, formation of aggregates and presence of stalks and crateriform structures. Phylogenetically, the strain belongs to the genus *Rubinisphaera*. Our analysis suggests that Pan54^T^ represents a novel species of this genus, for which we propose the name *Rubinisphaera italica* sp. nov. We suggest Pan54^T^ (= DSM 29369 = LMG 29789) as the type strain of the novel species.

## Introduction

Together with *Verrucomicrobia*, *Lentisphaerae*, *Kirimatiellaeota* and *Chlamydiae*, *Planctomycetes* form the medically and biotechnologically relevant PVC superphylum (Spring et al. 2016; Wagner and Horn 2006; Devos and Ward 2014). In the past, Planctomycetes were postulated as the missing link between bacteria and eukaryotes (Devos and Reynaud 2010) beyond the bacterial cell plan (Devos et al. 2013; Fuerst and Sagulenko 2011). This finding was based on proposed exceptional planctomycetal features, such as, lack of peptidoglycan (König et al. 1984), a compartmentalised cell plan (Lindsay et al. 1997), a nucleus-like structure (Fuerst and Webb 1991) and performance of endocytosis (Lonhienne et al. 2010). Further investigation of the planctomycetal physiology and morphology based on the advent of novel techniques changed this picture (Jogler et al. 2011; Jogler and Jogler 2013; Rivas-Marin et al. 2016). In particular, presence of peptidoglycan in some Planctomycetes was confirmed (Jeske et al. 2015; van Teeseling et al. 2015) and thus the cell plan of Planctomycetes was reinterpreted to be Gram-negative (Boedeker et al. 2017; Devos 2014a, b). But still, Planctomycetes remain exceptional and enigmatic in comparison to well-characterised canonical bacteria. They e.g. divide unusually, either by budding, binary fission or even a combination of both (Wiegand et al. 2019) and lack canonical divisome proteins including the otherwise universal FtsZ (Jogler et al. 2012; Pilhofer et al. 2008).

Many Planctomycetes survive in oligotrophic environments, such as seawater, by utilising a range of high-molecular-weight sugars derived from algae (Jeske et al. 2013; Lachnit et al. 2013) after attaching to these nutrient-rich surfaces (Bengtsson et al. 2012; Bondoso et al. 2015, 2014, 2017; Lage and Bondoso 2014; Vollmers et al. 2017). In this context, a specialised morphology including the unique pili-forming crateriform structures and an extremely enlarged periplasm might be involved in the uptake and cleavage of such polymeric compounds (Boedeker et al. 2017).

It is frequently observed that Planctomycetes are highly abundant in biofilms on nutrient-rich marine surfaces (Bengtsson and Øvreås 2010; Kohn et al. 2019b). This is astonishing when considering their moderate growth rates compared to faster-growing competitors in this ecological niche (Frank et al. 2014; Wiegand et al. 2018). It is thus likely that Planctomycetes are ‘talented’ producers of secondary metabolites, which could mediate (symbiotic) interactions with algae or act as antibiotics (Jeske et al. 2013). Gene clusters involved in small molecule production were predicted in planctomycetal genomes, which substantiates Planctomycetes as a promising source of such bioactive compounds (Graca et al. 2016; Wiegand et al. 2019).

Taken together, Planctomycetes are amongst the most maverick of all bacteria known thus far (Wiegand et al. 2018). We are steadily aiming to expand the collection of known Planctomycetes and recently presented 79 novel strains in an overview article (Wiegand et al. 2019). Here, we introduce and validly describe Pan54^T^, a novel planctomycetal strain that was isolated close to the volcanic island Panarea, and describe its morphology, physiology and phylogeny. The sampling location Panarea is located in the Tyrrhenian Sea, has an area of 3.3 km^2^ and is the second smallest of the Aeolian islands. The island itself is only a small part of a sub-marine edifice in form of a truncated cone with an eastern protrusion with its base being 1500 m below sea level (Gabbianelli et al. 1990). The entire cone has a diameter of 23 km and an area of 460 km^2^ and several thermal springs are located in proximity to the island. In the surroundings of Panarea areas with increased temperatures, higher levels of nutrients including nitrogen and sulfur sources are present, which motivated us to choose this geographical location as a valuable source of so far unknown species of prokaryotes (Gugliandolo et al. 2015).

## Material and methods

### Cultivation conditions and isolation

For strain isolation and cultivation M1H NAG ASW medium was used. For medium preparation 0.25 g peptone (Bacto), 0.25 g yeast extract (Bacto), 2.38 g (4-(2-hydroxyethyl)-1-piperazineethanesulfonic acid) (HEPES) (10 mM), 250 mL artificial sea water (ASW) and 20 mL sterile-filtered Hutner’s basal salt solution were mixed in a final volume of 973 mL double distilled water. The pH was adjusted to 7.5 using 5 M KOH and the solution was autoclaved for 20 min at 121 °C. After cooling, the following solutions were added aseptically: 1 mL of 25% (w/v) glucose, 5 mL vitamin solution, 1 mL trace element solution and 20 mL of a stock solution with 50 g/L *N*-acetyl glucosamine (NAG). ASW contained 46.94 g/L NaCl, 7.84 g/L Na_2_SO_4_, 21.28 g/L MgCl_2_ × 6 H_2_O, 2.86 g/L CaCl_2_ × 2 H_2_O, 0.384 g/L NaHCO_3_, 1.384 g/L KCl, 0.192 g/L KBr, 0.052 g/L H_3_BO_3_, 0.08 g/L SrCl_2_ × 6 H_2_O and 0.006 g/L NaF and was freshly prepared before addition to the base solution. Hutner’s basal salt solution was prepared by first dissolving 10 g nitrilotriacetic acid (NTA) in 700 mL double distilled water and adjusting the pH to 7.2 using 5 M KOH. After that, the following compounds were added: 29.7 g MgSO_4_ × 7 H_2_O, 3.34 g CaCl_2_ × 2 H_2_O, 0.01267 g Na_2_MoO_4_ × 2 H_2_O, 0.099 g FeSO_4_ × 7 H_2_O and 50 mL metal salt solution 44. The solution was filled up to 1 L, sterilised by filtering and stored at 4 °C. Metal salt solution 44 consisted of 250 mg/L Na_2_-EDTA, 1095 mg/L ZnSO_4_ × 7 H_2_O, 500 mg/L FeSO_4_ × 7 H_2_O, 154 mg/L MnSO_4_ x H_2_O, 39.5 mg/L CuSO_4_ × 5 H_2_O, 20.3 mg/L CoCl_2_ × 6 H_2_O and 17.7 mg/L Na_2_B_4_O_7_ × 10 H_2_O. In the first step, EDTA was dissolved and, if required, a few drops of concentrated H_2_SO_4_ were added to retard precipitation of heavy metal ions. Metal salt solution 44 was sterilised by filtration and stored at 4 °C. Vitamin solution contained per liter: 10 mg *p*-aminobenzoic acid, 4 mg biotin, 20 mg pyridoxine hydrochloride, 10 mg thiamine hydrochloride, 10 mg Ca-pantothenate, 4 mg folic acid, 10 mg riboflavin, 10 mg nicotinamide and 0.2 mg vitamin B12. *p*-Aminobenzoic acid was dissolved first and the solution was sterilised by filtration and stored in the dark at 4 °C. The trace element solution containing 1.5 g/L Na-nitrilotriacetate, 500 mg/L MnSO_4_ x H_2_O, 100 mg/L FeSO_4_ × 7 H_2_O, 100 mg/L Co(NO_3_)_2_ × 6 H_2_O, 100 mg/L ZnCl_2_, 50 mg/L NiCl_2_ × 6 H_2_O, 50 mg/L H_2_SeO_3_, 10 mg/L CuSO_4_ × 5 H_2_O, 10 mg/L AlK(SO_4_)_2_ × 12 H_2_O, 10 mg/L H_3_BO_3_, 10 mg/L NaMoO_4_ × 2 H_2_O and 10 mg/L Na_2_WO_4_ × 2 H_2_O was sterilised by filtration and stored in the dark at 4 °C.

Strain Pan54^T^ was isolated on the 10th of September 2013 from an algal surface in hydrothermal area 26 (sampling site 38.6392 N, 15.1051 E) close to the island Panarea in the north of Sicily, Italy. Algae leaves were initially washed with 0.5 × artificial sea water (ASW) and placed on M1H ASW solid medium (lacking NAG) containing 8 g/L gelrite additionally supplemented with 20 mg/L cycloheximide, 1000 mg/L streptomycin and 200 mg/L ampicillin. Two different pH values (6.5 and 8.0) were tested and plates were cultivated at 20 °C for 2–3 weeks. Isolated colonies were then streaked on a new plate and maintained in liquid M1H NAG ASW medium. Initial amplification and sequencing of the 16S rRNA gene was performed as previously described (Rast et al. 2017).

### Deposition of genomic data

Genome data (acc. no. SJPG00000000) and the 16S rRNA gene sequence (acc. no. MK554545) were deposited in the GenBank database.

### Light microscopy

Phase contrast (Phaco) analyses were performed employing a Nikon Eclipse Ti inverted microscope with a Nikon DS-Ri2 camera (blue LED). Specimens were immobilised in MatTek glass bottom dishes (35 mm, No. 1.5) employing a 1% agarose cushion (Will et al. 2018). Images were analysed using the Nikon NIS-Elements software (version 4.3). To determine the cell size, at least 100 representative cells were counted manually (Annotations and Measurements, NIS-Elements) or by using the NIS-Elements semi-automated object count tool (smooth: 4×, clean: 4×, fill holes: on, separate: 4×).

### Electron microscopy

For field emission scanning electron microscopy (FESEM) bacteria were fixed in 1% (v/v) formaldehyde in HEPES buffer (3 mM HEPES, 0.3 mM CaCl_2_, 0.3 mM MgCl_2_, 2.7 mM sucrose, pH 6.9) for 1 h on ice and washed once employing the same buffer (Rast et al. 2017). Cover slips with a diameter of 12 mm were coated with a poly-l-lysine solution (Sigma-Aldrich) for 10 min, washed in distilled water and air-dried. 50 µL of the fixed bacteria solution was placed on a cover slip and allowed to settle for 10 min. Cover slips were then fixed in 1% glutaraldehyde in TE buffer (20 mM TRIS, 1 mM EDTA, pH 6.9) for 5 min at room temperature and subsequently washed twice with TE buffer before dehydrating in a graded series of acetone (10, 30, 50, 70, 90, 100%) on ice for 10 min at each concentration. Samples from the 100% acetone step were brought to room temperature before placing them in fresh 100% acetone. Samples were then subjected to critical-point drying with liquid CO_2_ (CPD 300, Leica). Dried samples were covered with a gold/palladium (80/20) film by sputter coating (SCD 500, Bal-Tec) before examination in a field emission scanning electron microscope (Zeiss Merlin) using the Everhart Thornley HESE2 detector and the inlens SE detector in a 25:75 ratio at an acceleration voltage of 5 kV. Transmission electron microscopy (TEM) was performed as described before (Kohn et al. 2016).

### Physiological analyses

For determination of the pH optimum 100 mM HEPES was used for cultivations at pH 7.0, 7.5 and 8.0. For cultivation at pH 5.0 and 6.0 HEPES was replaced by 100 mM 2-(*N*-morpholino)ethanesulfonic acid (MES), whereas 100 mM *N*-cyclohexyl-2-aminoethanesulfonic acid (CHES) served as a buffering agent at pH 9.0 and 10.0. Cultivations for determination of the pH optimum were performed at 28 °C. For determination of the temperature optimum Pan54^T^ was cultivated in M1H NAG ASW medium at pH 7.5 at different temperatures ranging from 10 to 40 °C. Fatty acid composition of Pan54^T^ was analysed based on a protocol described previously (Kohn et al. 2016).

### Phylogenetic analyses

The genome of strain Pan54^T^ was published previously (Wiegand et al. 2019) and is available from GenBank under acc. no. SJPG00000000. The GenBank acc. no. of the 16S rRNA gene is MK554545. 16S rRNA gene phylogeny was computed for strain Pan54^T^, the type strains of all described planctomycetal species (May 2019) and all isolates recently published (Wiegand et al. 2019). The 16S rRNA gene sequences were aligned with SINA (Pruesse et al. 2012). The phylogenetic analysis was done employing a maximum likelihood (ML) approach with 1,000 bootstraps, the nucleotide substitution model GTR, gamma distribution and estimation of proportion of invariable sites (GTRGAMMAI option) (Stamatakis 2014). Three 16S rRNA genes of bacterial strains from the PVC superphylum served as outgroup. The *rpoB* nucleotide sequences (encoding the RNA polymerase β-subunit) were taken from publicly available genome annotations and the sequence identities were determined as described previously (Bondoso et al. 2013) with Clustal Omega (Sievers et al. 2011) alignment and matrix calculation upon extracting only those parts of the sequence that would have been sequenced with the described primer set. The average nucleotide identity (ANI) was calculated using OrthoANI (Lee et al. 2016). The average amino acid identity (AAI) was gained with the aai.rb script of the enveomics collection (Rodriguez-R and Konstantinidis 2016). The percentage of conserved proteins (POCP) was calculated as described before (Qin et al. 2014).

## Results and discussion

### Phylogenetic analysis

**Fig. 1:**
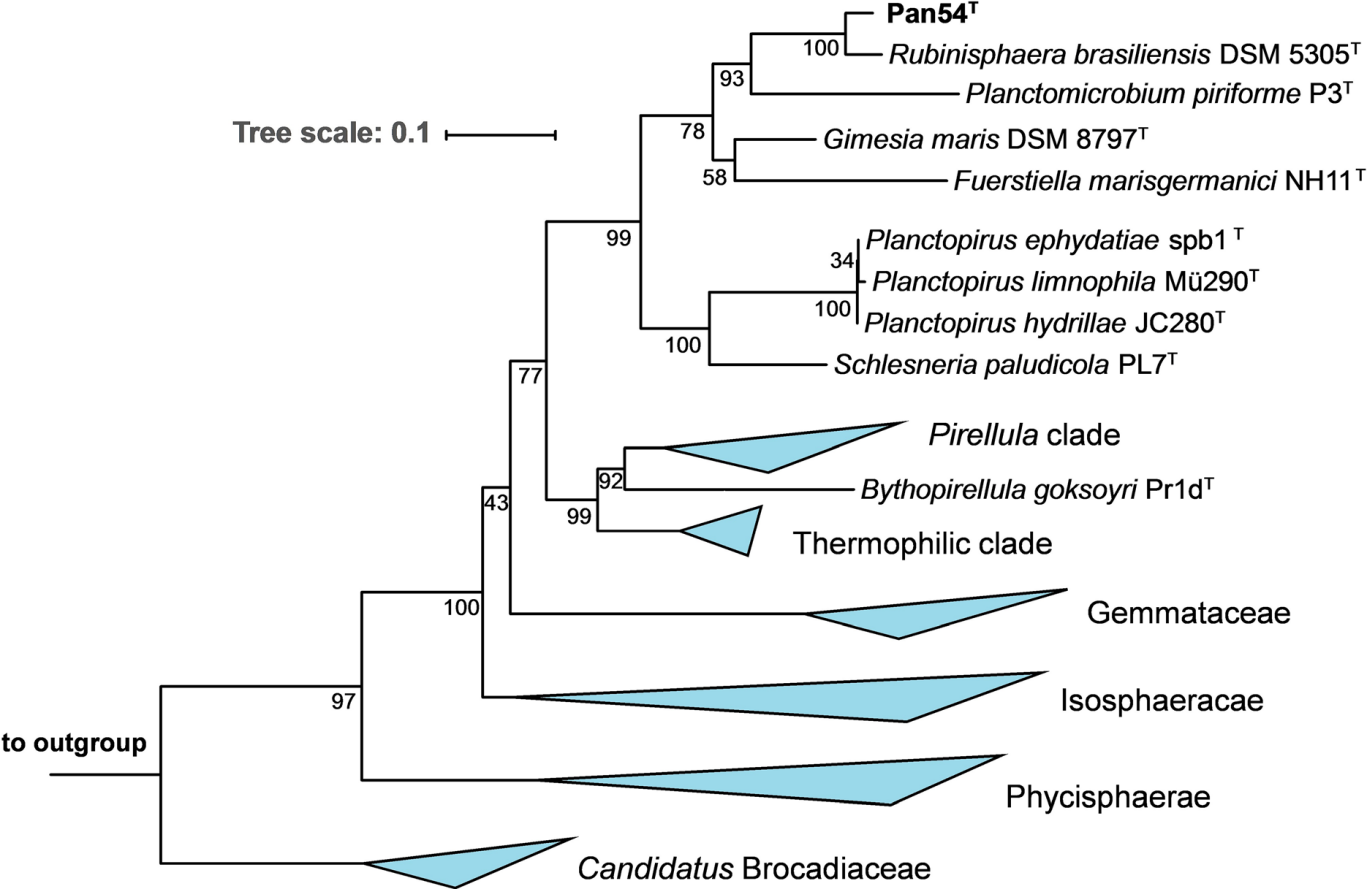
Phylogenetic analysis. The phylogenetic tree highlighting the position of Pan54^T^ is depicted. 16S rRNA gene phylogeny was computed using the maximum likelihood method. Bootstrap values after 1000 resamplings (in %) are given at the nodes. The outgroup consists of three 16S rRNA genes from the PVC superphylum. The “*Pirellula* clade” includes species of the genera *Rhodopirellula*, *Rubripirellula*, *Roseimaritima*, *Mariniblastus*, *Pirellula* and *Blastopirellula*. The “Thermophilic clade” comprises the genera *Thermostilla*, *Thermogutta* and *Thermopirellula*

In our phylogenetic analysis Pan54^T^ appears in a monophyletic clade with its closest relative *Rubinisphaera brasiliensis* DSM 5305^T^ (Fig. 1). *R. brasiliensis* DSM 5305^T^ was originally isolated from a water sample of Lagoa Vermelha, a salt pit near Rio de Janeiro, Brasil, initially described as “*Planctomyces brasiliensis”* in 1989 and was later reclassified (Scheuner et al. 2014; Schlesner 1989). *R. brasiliensis* is currently the only validly described species within the genus *Rubinisphaera*. Pan54^T^ and *R. brasiliensis* DSM 5305^T^ share a 16S rRNA gene sequence identity of 96.2%. This value is below the threshold of 98.7% for a novel species (Stackebrandt and Ebers 2006), but above the threshold for a novel planctomycetal genus of 94.5% (Yarza et al. 2014). Both strains share 76.5% *rpoB* gene identity, which is below the 96.3% cutoff proposed to distinguish between different planctomycetal species and above the threshold for novel genera of 72% sequence identity (Bondoso et al. 2013). *Average nucleotide identity* (ANI) between both strains is 69.7% and distinctly below the species threshold of 95 - 96% for ANIb (ANI calculated with the BLAST algorithm) (Kim et al. 2014). The *average amino acid identity* (AAI) between Pan54^T^ and its relative *R. brasiliensis* DSM 5305^T^ is 58.1% and thus fits in the range for species belonging to the same bacterial genus (Rodriguez et al. 2018). Finally, the *percentage of conserved proteins* (POCP) of 62.3% between both strains also indicates that they belong to the same genus, as a prokaryotic genus is proposed to be formed by a group of species with pairwise POCP values higher than 50% (Qin et al. 2014). Close relatives apart from *R. brasiliensis* DSM 5305^T^ include *Planctomicrobium piriforme* P3^T^ (Kulichevskaya et al. 2015), *Gimesia maris* DSM 8797^T^ (Scheuner et al. 2014), *Fuerstiella marisgermanici* NH11^T^ (formerly designated “*Fuerstia marisgermanicae*”) (Kohn et al. 2019a, 2016) and species of the *Planctopirus* genus (Fig. 1).

### Morphological, physiological and biochemical analyses

**Fig. 2:**
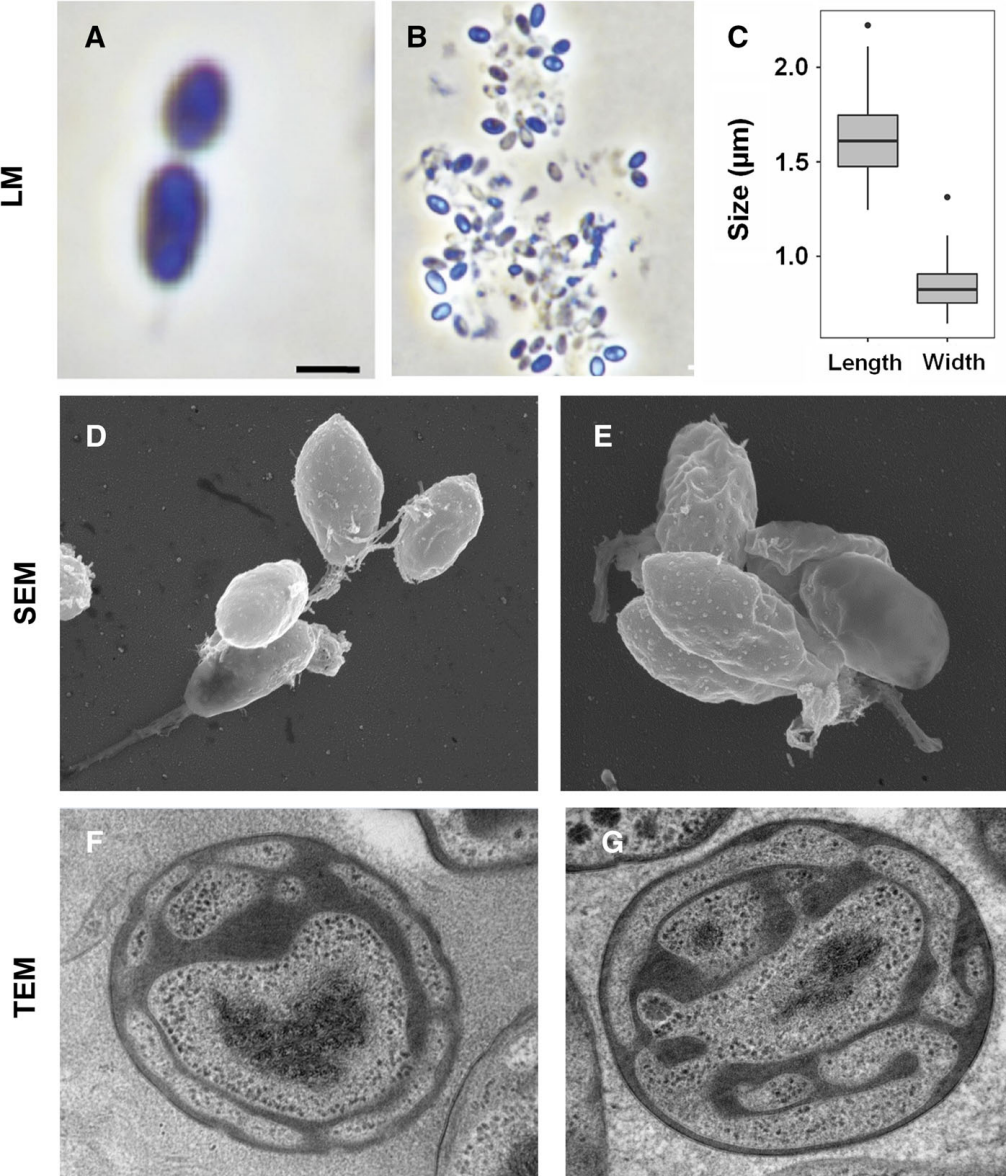
Microscopy images and cell size plot of Pan54^T^. Pictures from light microscopy (LM) (**a**, **b**), scanning electron microscopy (SEM) (**d**, **e**) and transmission electron microscopy (TEM) (**f**, **g**) are shown. The scale bars are 1 µm. For determination of the cell size (**c**) at least 100 representative cells were counted manually or by using a semi-automated object count tool during scanning electron microscopy

For a morphological characterization, Pan54^T^ cells were harvested during the exponential growth phase. Pan54^T^ cells were found to be pear-shaped (1.6 ± 0.2 µm × 0.8 ± 0.1 µm) (Fig. 2a–c) and form strong aggregates and biofilms. Cells have a textured surface and contain evenly distributed crateriform structures (Fig. 2d, e). Pan54^T^ divides by polar budding (Fig. 2a). Daughter cells have the same shape as mother cells. Thin sections of Pan54^T^ cells show a condensed nucleoid and cytoplasmic invaginations (Fig. 2f, g). Colonies are white indicating a lack of carotenoids as pigmenting compounds. Detailed information on morphology, locomotion and cell division is summarised in Table 1. Pan54^T^ has a very similar morphology as *R. brasiliensis*. In the direct comparison Pan54^T^ has a slightly elongated shape and crateriform structures appear to be more pronounced. Similar to *R. brasiliensis* rosette formation was observed rather than formation of larger aggregates (Scheuner et al. 2014). The colony colour of non-pigmented Pan54^T^ differs from the yellow to orange pigmentation of *R. brasiliensis*.

**Table 1:** Phenotypic and genotypic features of Pan54^T^ in comparison to *R. brasiliensis* DSM 5305^T^ (Scheuner et al. 2014; Schlesner 1989)

Characteristics	Pan54^T^	*R. brasiliensis* DSM 5305^T^
*Phenotypic features*		
Color	White	Yellow to orange
Size	1.6 × 0.8 µm	0.7 – 1.8 µm
Shape	Pear-shaped	Spherical to ovoid
Aggregates	Yes	Yes
Division	Budding	Budding
Dimorphic life cycle	n.o.	Yes
Flagella	n.o.	Yes
Crateriform structures	Yes, overall	Yes
Fimbriae	Yes, polar matrix or fiber	Yes
Capsule	n.o.	n.o.
Bud shape	Like mother cell	Like mother cell
Budding pole	Polar	Polar
Stalk	Yes	Yes
Holdfast structure	n.o.	Yes
*Genotypic features*		
Genome size [bp]	6,704,479	6,006,602
Plasmids [bp]	n.o.	n.o.
GC [%]	48.8 ± 0.5	56.4
Completeness [%]	96.55	94.83
Contamination [%]	1.88	3.45
Protein-coding genes	5275	4824
Hypothetical proteins	2229	2581
Protein-coding genes/Mb	787	803
Coding density [%]	85.4	86.1
16S rRNA genes	2	2
tRNA genes	58	50

*n.o.* not observed, *n/a* not available

**Fig. 3:**
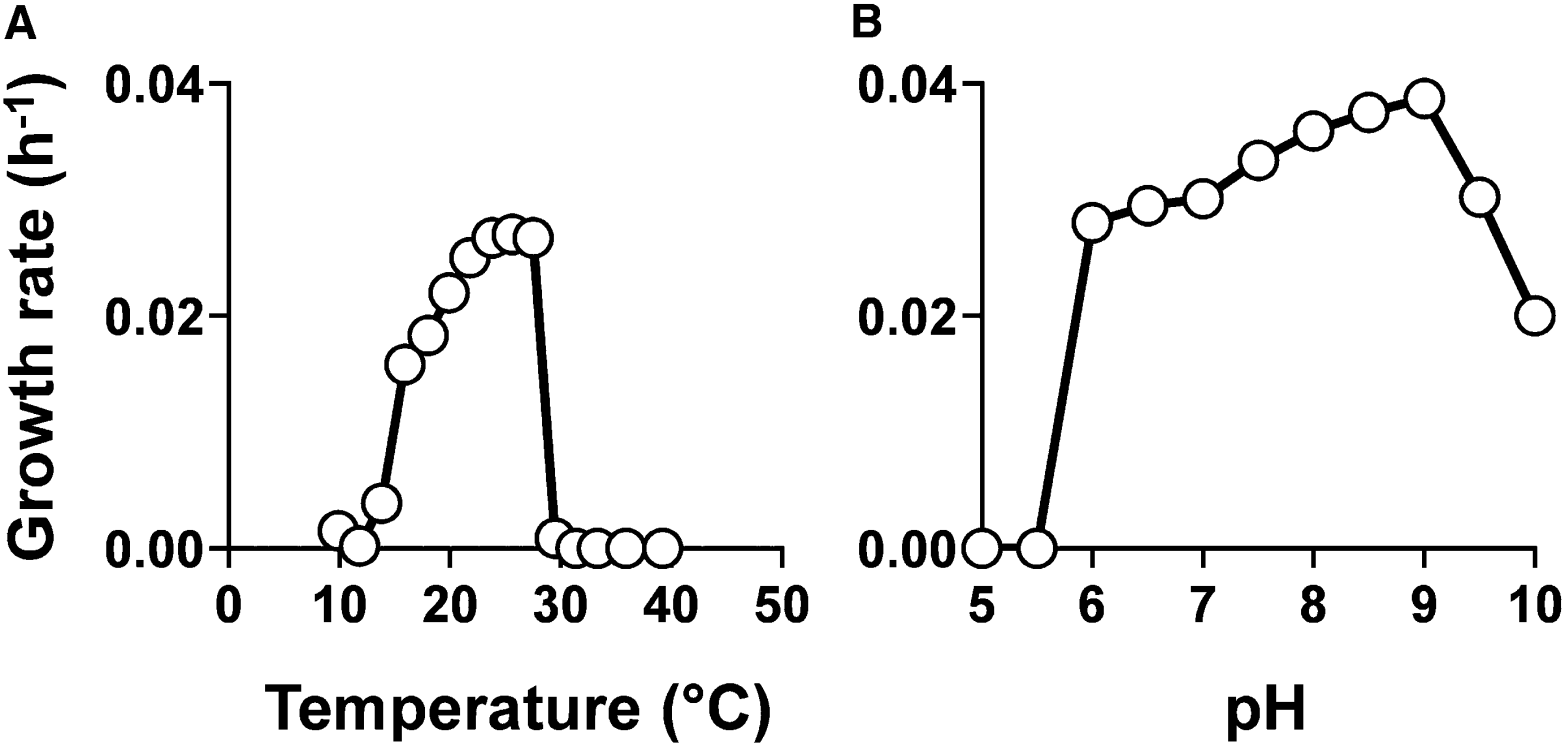
Temperature and pH optimum of strain Pan54^T^. Data points show average growth rates obtained after cultivation in M1H NAG ASW medium in biological triplicates. Cultivations at different temperatures (**a**) were performed at pH 7.5. Cultivations at different pH values (**b**) were conducted at 28 °C

In physiological experiments, Pan54^T^ grew at a temperature range of 14–27 °C (Fig. 3a) and a pH range of 6.0–10.0 (Fig. 3b), but failed to grow at 30 °C or above (Fig. 3a). The optimal conditions turned out to be pH 9.0 and 26 °C. The observed temperature optimum of Pan54^T^ is lower compared to *R. brasiliensis* (30–33 °C) (Schlesner 1989). *R. brasiliensis* even grows at temperatures of 37 °C (Schlesner 1989), while Pan54^T^ failed to grow at 30 °C or higher. Taken together, growth of Pan54^T^ is mesophilic and slightly alkaliphilic. A maximal growth rate of 0.039 h^−1^ was observed under the given conditions corresponding to a generation time of 18 h (Fig. 3). This value is in the range of 0.01–0.09 h^−1^ (generation times of 8–70 h), which we typically observed for planctomycetal strains isolated and characterised in our lab so far.

Major fatty acids of Pan54^T^ include summed feature 3 (palmitoleic acid (16:1 ω7c), 15:0 iso 2-OH) (57%), palmitic acid (16:0) (36%) and *cis*-vaccenic acid (18:1 ω7c) (5%) (Table 2). The composition is similar to *R. brasiliensis* in which 16:0, 16:1 and 18:1 were also found as the major fatty acids (Scheuner et al. 2014). However, the composition appears to be more restricted to these three in Pan54^T^ as other fatty acid were only detected in traces (< 0.5%) in this strain.

**Table 2:** Fatty acid composition of Pan54^T^

Fatty acid	Share in %
12:0 3-OH	0.06
14:1 ω5c	0.10
14:0	0.21
15:1 ω6c	0.13
15:0	0.26
Summed feature 3 (16:1 ω7c, 15:0 iso 2-OH)	56.53
16:1 ω5c	0.21
16:0	35.82
17:1 ω8c	0.15
17:0	0.10
18:1 ω9c	1.22
18:1 ω7c	4.73
18:0	0.37
20:1 ω7c	0.11

### Genomic characteristics

The genome of Pan54^T^ has a size of 6.7 Mb and is slightly larger compared to *R. brasiliensis* (6.0 Mb), whereas the GC content is lower (48.8% for Pan54^T^, 56.4% for *R. brasiliensis*). Relevant genome characteristics are summarised in Table 1. Automated gene prediction and annotation identified 5275 putative protein-encoding genes, of which 42% (2229 genes) are annotated as hypothetical proteins. The calculated values correspond to 787 protein-coding genes per Mb and a coding density of 85%. Except for differences in GC content no striking differences between genome features of Pan54^T^ and *R. brasiliensis* DSM 5305^T^ were observed (Table 1). The number of 16S rRNA in both strains is identical and number of tRNAs is very similar.

## Conclusion

Based on the data collected during strain characterisation, Pan54^T^ represents a novel species within the genus *Rubinisphaera.* We propose the name *Rubinisphaera italica* sp. nov. and present Pan54^T^ as the type strain of the species.

### Emended description of the genus *Rubinisphaera* Scheuner et al. (2014)

The description of the genus *Rubinisphaera* given previously (Scheuner et al. 2014), with the following modification: The GC content is between 48 and 57%.

### Description of *Rubinisphaera italica* sp. nov.

*Rubinisphaera italica* (i.ta'li.ca. L. fem. adj. *italica* of Italy; corresponding to the isolation of the strain from Italy). Cells are pear-shaped (length: 1.6 ± 0.2 µm, width: 0.8 ± 0.1 µm), form aggregates and divide by polar budding. Cells grow at ranges of 14–27 °C (optimum 26 °C) and pH 6.0–10.0 (optimum 9.0). Colonies are white. The genome (acc. no. SJPG00000000) and 16S rDNA sequence (acc. no. MK554545) are available from the GenBank database. The genome has a GC content of 48.8% and is 6.70 Mb in length. The proposed type strain is Pan54^T^ (DSM 29369 = LMG 29789) isolated from an algal surface at a hydrothermal area close to Panarea, Italy.
